# Serological and molecular characteristics of pathogenic *Leptospira* in rodent populations in Fujian Province, China, 2018–2020

**DOI:** 10.1186/s12866-022-02566-2

**Published:** 2022-06-07

**Authors:** Guoying Xu, Haiyan Qiu, Weijun Liu, Xiugao Jiang, Yung-Fu Chang, Jiaxiong Wang, Zhenpeng Li, Yongzhang Zhu, Cuicai Zhang, Fangzhen Xiao

**Affiliations:** 1 Fujian Provincial Key Laboratory of Zoonosis Research, Fujian Center for Disease Control and Prevention, Fuzhou, China; 2 College of Public Health, Fujian Medical University, Fuzhou, China; 3 State Key Laboratory of Infectious Disease Prevention and Control, National Institute for Communicable Disease Control and Prevention, Chinese Center for Disease Control and Prevention, Beijing, China; 4 Collaborative Innovation Center for Diagnosis and Treatment of Infectious Diseases, Hangzhou, China; 5 Department of Population Medicine and Diagnostic Sciences, College of Veterinary Medicine, Cornell University, Ithaca, NY USA; 6 School of Global Health, Chinese Center for Tropical Diseases Research, Shanghai Jiao Tong University School of Medicine, Shanghai, China

**Keywords:** *Leptospira*, Rodent, Genetic diversity, Serological characteristics

## Abstract

**Background:**

Leptospirosis is a significant emerging infectious disease worldwide. Rodents are considered to be the most critical hosts of *Leptospira spp.* Fujian Province is a region highly endemic for leptospirosis in China. However, the genetic diversity of leptospires circulating among rodents in Fujian is limited.

**Results:**

The carrier status of rodents for *Leptospira spp.* was investigated by culture and serological detection in Fujian during 2018–2020. A total of 710 rodents, including 11 species, were trapped, with *Rattus losea* being the dominant trapped species (50.56%). Fourteen pathogenic *Leptospira* strains were obtained. Seven *L. borgpetersenii* serogroup Javanica strains belonging to ST143, 4 *L. interrogans* serogroup Icterohaemorrhagiae strains belonging to ST1 and ST17, 2 *L. interrogans* serogroup Bataviae strains belonging to ST96 and ST333, and 1 *L. interrogans* serogroup Pyrogenes strains belonging to ST332 were identified using 16S rDNA gene sequencing, microscopic agglutination test (MAT) and Multilocus sequence typing (MLST). *L. borgpetersenii* serogroup Javanica belonging to ST143 was the dominant type (50.00%). A total of 387 rodent serum samples were tested by MAT. Serum were considered positive for seroreactivity at a titer ≥ 1:160 against at least one serovar. A total of 90 (23.26%) serum samples tested positive, and four serogroups were identified, with Javanica being the dominant serogroup (87.78%), which was similar to the dominant serogroup isolated from rodents. This study demonstrates a high prevalence of leptospirosis in rodents and public health education among high-risk workers is highly recommended.

**Conclusions:**

*R. losea* was the dominant trapped rodent, and *L. borgpetersenii* serogroup Javanica ST143 was widely distributed among rodents in Fujian from 2018 to 2020. Despite the low number of isolates obtained from rodents, this study suggests that continuous epidemiological surveillance of the aetiological characteristics of pathogenic *Leptospira* in wild animal reservoirs may help reduce the possible risk of disease transmission.

**Supplementary Information:**

The online version contains supplementary material available at 10.1186/s12866-022-02566-2.

## Background

As one of the most widespread zoonotic diseases, leptospirosis is caused by pathogenic spirochetes of the *Leptospira* genus, and an estimated one million human leptospirosis cases and 58,900 deaths occur annually [1–3]. The genus *Leptospira* is classified into pathogenic, intermediate, and saprophytic species, and more than 300 serovars have been reported based on antigenic similarity [4, 5]. *L. interrogans*, *L. borgpetersenii* and *L. kirschneri* are the main pathogenic species of leptospirosis in animals and humans worldwide [6–8].

Humans and animals can be infected primarily through direct or indirect exposure to urine or tissues of infected mammalian hosts and urine-contaminated soil or water [9]. Among wildlife species, rodents are considered the most critical reservoir of pathogenic *Leptospira* worldwide due to their close contact with humans, infected livestock and companion animals [1, 10]. Therefore, long-term active surveillance and investigations of the carriage status of rodents will contribute to understanding animal-to-human transmission, investigating outbreaks, and tracking sources of leptospirosis and will be instrumental in designing better bacterin vaccines for leptospirosis. Although leptospirosis has significantly decreased over the past few years worlwide, leptospirosis is still recognized as a critical zoonosis in China. Based on an investigation from 2018 to 2020 in Fujian Province, the leptospirosis disease incidence was between 0.0381 and 0.0639 cases per 100,000. This incidence was slightly higher than the national incidence (between 0.0113 and 0.0212 cases per 100,000 during 2018–2020), showing that Fujian Province was a significant epidemic region for leptospirosis (data are available from the National Notifiable Infectious Disease Surveillance System in China with login authorization: https://10.249.6.18:8881/cdc/login). The average incidence rates in some regions of Fujian Province, especially Pucheng (0.334 cases per 100,000), Luoyuan (0.778 cases per 100,000), and Wuping (0.361 cases per 100,000) city in Fujian, are markedly higher than the national incidence in China (0.0159 cases per 100,000). Unfortunately, people living in Fujian Province have a poor level of knowledge about potential risk factors for leptospirosis. Our previous study reported two predominant pathogenic *Leptospira* named *L. interrogans* serogroup Icterohaemorrhagiae and *L. borgpetersenii* serogroup Javanica, with sequence type (ST) 1 and ST143 being the dominant STs circulating in rodent populations in Jiangxi Province in China [11]. However, there is little information regarding the most crucial rodent hosts and transmission to humans in the highly endemic district of Fujian Province. Therefore, this study aimed to investigate the prevalence of pathogenic *Leptospira* in potential reservoir animals of rodents and to identify the predominant species/serogroups/genotypes circulating in rodent populations in Fujian Province. The detailed molecular and serological characteristics of *Leptospira* in this area will improve diagnoses and provide deeper insights into the molecular characterization, epidemiology, and prevention and control of leptospirosis.

## Results

### Rodent distribution from Fujian Province

A total of 9,136 traps were placed, and 710 rodents belonging to 11 different species were successfully captured between 2018 and 2020 in Fujian Province. The species identifications of the trapped rodents and the numbers of rodents with positive cultures are listed in Table 1. The density of rodents was between 4.81–16.77% in the six trapping sites in Fujian Province, with Luoyuan having the highest density of rodents (16.77%). The most abundantly trapped species was *R. losea* (50.56%, 359/710), followed by *N. fulvescens* (19.86%, 141/710) and *R. flavipectus* (18.17%, 129/710) in Fujian.

**Table 1:** Rodent distribution and numbers of rodents with positive renal cultures during 2018–2020 in Fujian Province

regions	A	B	Rodent density	*Rattus losea*	*Rattus flavipectus*	*Rattus norvegicus*	*Niviventer fulvescens*	*Berylmys bowersi*	*Niviventer niviventer*	*house mouse*	*Apodemus agrarius*	*Niviventer coninga*	*Microtus fortis*	*Bandicota indica*	Strains
Changtai	1777	100	5.63	0/34	2/55	0/9	0/1	0/0	0/0	0/1	0/0	0/0	0/0	0/0	2
Fuqing	561	27	4.81	1/24	0/0	0/0	0/2	0/0	0/1	0/0	0/0	0/0	0/0	0/0	1
Luoyuan	483	81	16.77	1/76	0/1	0/0	0/0	0/0	0/2	0/0	0/0	0/0	0/0	0/2	1
Jianyang	378	37	9.79	0/6	0/0	0/0	1/25	0/2	1/1	0/0	0/0	0/1	0/0	0/2	2
Pucheng	315	37	11.75	0/2	0/0	0/0	0/9	0/0	0/7	0/0	2/11	0/2	0/6	0/0	2
Wuping	5622	428	7.61	3/217	3/73	0/11	0/104	0/23	0/0	0/0	0/0	0/0	0/0	0/0	6
Total	9136	710	7.77	5/359	5/129	0/20	1/141	0/25	1/11	0/1	2/11	0/3	0/6	0/4	14

A-Number of total traps placed for each region

B-Number of rodents trapped for each region

Rodent density calculated as (B/A * 100)

Fisher’s exact test revealed highly significant differences in the species distribution of trapped rodents across different collected sites (*P* < 0.001) (Table S1). These species represented most of the trapped rodent diversity in Fujian Province. *R. losea* was the most commonly trapped species in Fuqing (24/27, 88.89%), Luoyuan (76/81, 93.83%), and Wuping (217/428, 50.70%), *R. flavipectus* was most common in Changtai (55/100, 55.0%), *N. fulvescens* was most common in Jianyang (25/37, 67.57%), and *A. agrarius* was most common in Pucheng (11/37, 29.73%).

### Leptospiral isolation from Fujian Province

A total of 14 strains from 5 different species of field rodents were isolated (Table 1 and Table S2). The carriage rates of these 5 different species of rodents based on culture isolation were between 0.71–18.18%, with the highest rates in *A. agrarius* (2/11, 18.18%), followed by *N. niviventer* (1/11, 9.09%), *R. flavipectus* (5/129, 3.88%), *R. losea* (5/359, 1.39%) and *N. fulvescens* (1/141, 0.71%), indicating that *A. agrarius* may be the primary trapped carrier of *Leptospira* in Fujian.

### Results of species identification of 14 isolates based on 16S rDNA gene sequencing

**Fig. 1:**
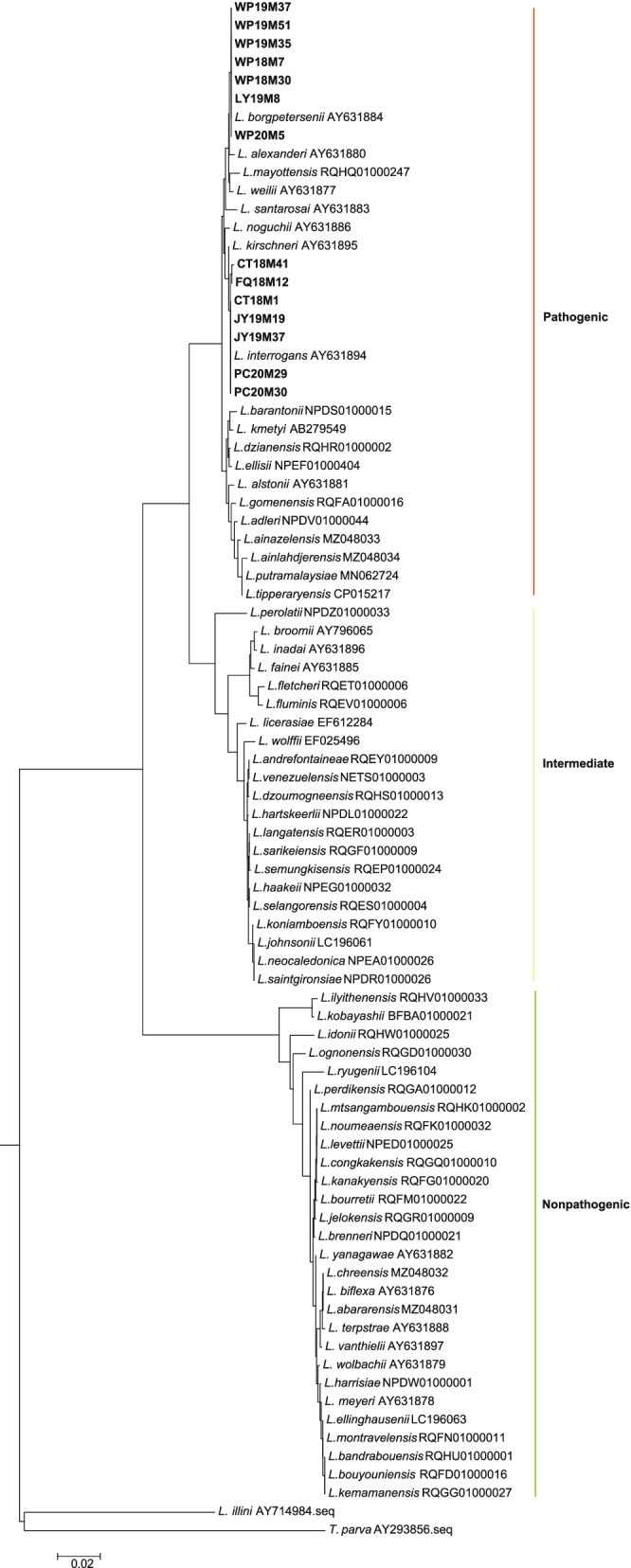
Phylogenetic analysis based on the 16S rDNA gene for the 14 pathogenic *Leptospira* strains in Fujian. Two species, *L. interrogans* and *L. borgpetersenii,* were identified among these 14 *Leptospira* strains

Among the 14 isolates, species identification revealed that *L. interrogans* and *L. borgpetersenii* accounted for 50%, respectively (Fig. 1 and Table S2). Fisher’s exact test revealed highly significant differences in the distribution of leptospiral species prevalence across the collected sites among the 14 strains (*p* < 0.001) (Table S3), but no differences across rodent species (Table S4). *L. interrogans* was widely represented in Jianyang, Pucheng, Fuqing and Changtai, while *L. borgpetersenii* was only distributed in the remaining two cities of Luoyuan and Wuping (Table S3). Furthermore, *L. interrogans* and *L. borgpetersenii* were present every year between 2018 and 2020 (Table S2).

### Serogroup identification of 14 isolates from Fujian Province from 2018 to 2020

A total of four serogroups were identified among these 14 isolates. Seven *L. interrogans* included 4 (4/14, 28.57%) serogroup Icterohaemorrhagiae, 2 (2/14, 14.29%) Bataviae and 1 (1/14, 7.14%) Pyrogenes. All of the remaining seven *L. borgpetersenii* (7/14, 50%) belonged to Serogroup Javanica. Serogroups Javanica and Icterohaemorrhagiae were the most prevalent serogroups (Table S2).

There were some significant regional differences across the six collected sites in the distribution of the dominant serogroups among the 14 strains according to Fisher’s exact test (*p* < 0.05) (Table S5), but there were no differences across rodent species (Table S6). Icterohaemorrhagiae was the only predominant serogroup in Jianyang and Pucheng, Bataviae in Fuqing, and Javanica in Luoyuan and Wuping, and Serogroups Bataviae and Pyrogenes were identified in Changtai. In addition, serogroup Javanica was present every year between 2018 and 2020 in Wuping (Table S2).

### Genetic diversity of 14 isolates from Fujian using MLST analysis

**Fig. 2:**
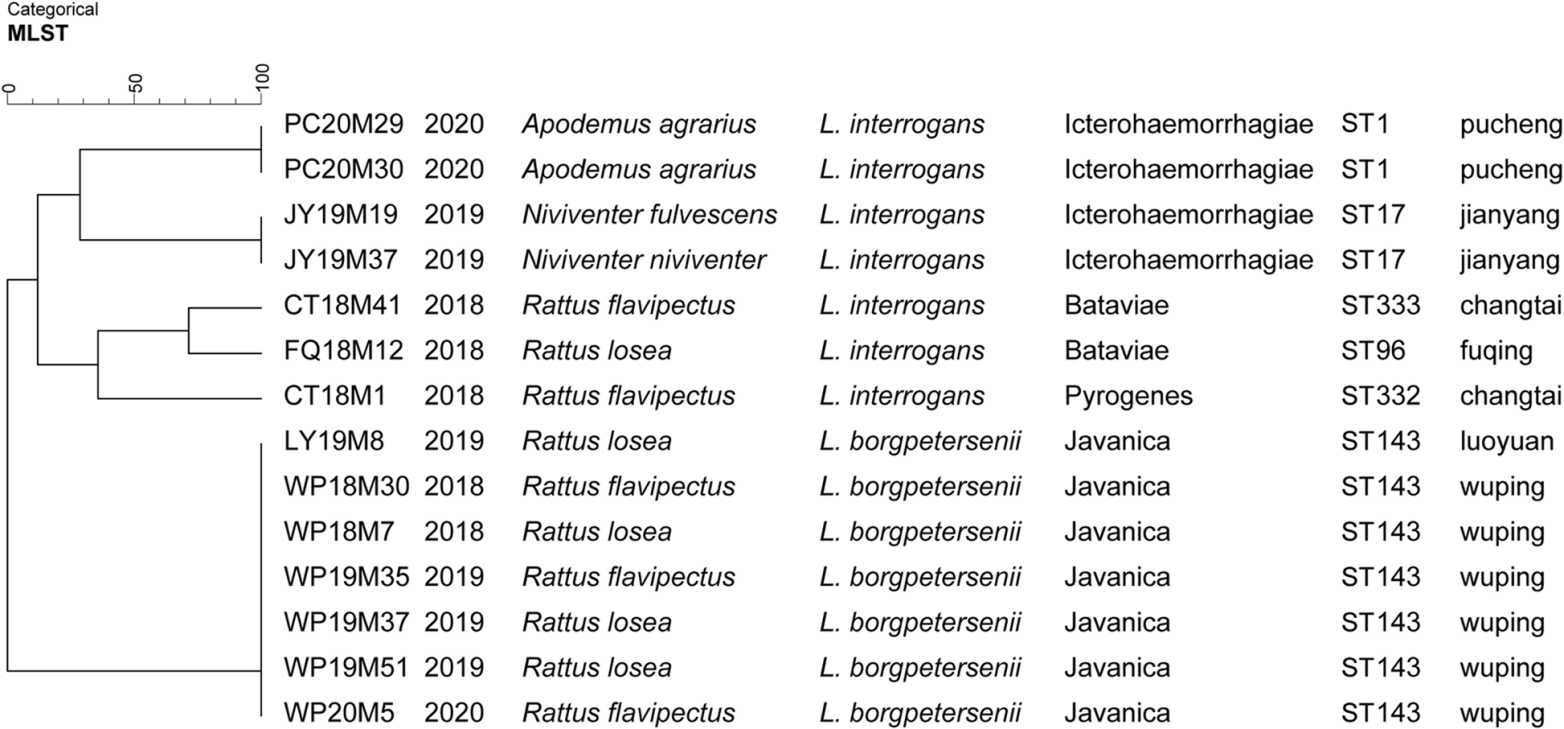
UPGMA dendrogram indicating the diversity of 14 pathogenic *Leptospira* strains isolated from Fujian Province in MLST analysis. Groups were defined by a similarity of 60%. The dendrogram reveals that the 14 pathogenic *Leptospira* strains belonged to five major clades

A total of 6 different STs, including 2 new STs (ST332 and ST333), were identified from 14 pathogenic *Leptospira* isolates in Fujian Province (Table S2). The most predominant ST was *L. borgpetersenii* ST143 (7/14), followed by *L. interrogans* ST1 (2/14) and *L. interrogans* ST17 (2/14). The remaining 3 isolates belonged to 3 different STs (Table S2). The UPGMA dendrogram of STs showed that the strains isolated from the same trapping sites clustered closely together except for the two strains belonging to two different STs from Changtai (Fig. 2). The dendrogram revealed that the 14 pathogenic *Leptospira* strains belonged to five major clades. Fisher’s exact test revealed highly significant differences in the distribution of ST prevalence across collected sites/host species among the 14 strains (*p* < 0.01) (Table S7 and Table S8). ST143 was distributed in *R. flavipectus* and *R. losea,* ST1 was distributed in *A. agrarius,* ST17 was distributed in *N. fulvescens* and *N. nivivente,* and ST322 and ST323 were distributed in *R. flavipectus* and ST96 in *R. losea*. In addition, there were some significant regional variations in the distribution of the dominant STs. ST143 was the only typical ST in Luoyuan and Wuping, ST17 was identified in Jianyang, ST1 was identified in Pucheng, and ST96 was identified in Fuqing, while ST332 and ST333 were identified in Changtai (Fig. 2).

### MAT of rodent sera

Three hundred twenty-three rodents had insufficient serum. Therefore, a total of 387 rodent serum samples from the trapped rodents were tested for *Leptospira* antibodies by MAT with a panel of 10 local serovars. A total of 90 (90/387, 23.26%) serum samples were tested positive at a MAT titer ≥ 1:160 and four leptospiral serogroups were identified (Table 2 and Table S9). The major circulating leptospiral serogroup was Javanica (79/90, 87.78%), followed by Pomona (7/90, 7.78%), Australis (2/90, 2.22%) and Icterohaemorrhagiae (2/90, 2.22%), with positive agglutination titres varying between 160 and 2560 (Table S9). The highest titer detected was 1:2,560 for serogroups Javanica and Pomona, followed by the titer of 1:1,280 for Javanica, Pomona, and Icterohaemorrhagiae.

**Table 2:** Seroprevalence of pathogenic *Leptospira*l serogroups among rodents samples by microscopic agglutination test

Collected Locations	Numbers	Positive(n)	Prevalence(%)	Icterohaemorrhagiae	Javanica	Autumnalis	Australis	Pomona	Hebdomadis	Grippotyphosa	Pyrogenes	Bataviae	Sejroe
Changtai	47	3	6.38	0	2	0	0	1	0	0	0	0	0
Wuping	180	54	30	0	48	0	2	4	0	0	0	0	0
Fuqing	26	11	42.31	0	9	0	0	2	0	0	0	0	0
Luoyuan	67	19	28.36	0	19	0	0	0	0	0	0	0	0
Jianyang	36	1	2.78	0	1	0	0	0	0	0	0	0	0
Pucheng	31	2	6.45	2	0	0	0	0	0	0	0	0	0
Total	387	90	23.26	2	79	0	2	7	0	0	0	0	0

Seroprevalence varied significantly across the six regions, with the highest prevalence in Fuqing (11/26, 42.31%), followed by Wuping (54/180, 30.00%), Luoyuan (19/67, 28.36%), Pucheng (2/31, 6.45%), Changtai (3/47, 6.38%), and Jianyang (1/36, 2.78%) (*P* < 0.001) (Table S10). Serogroup Javanica was the major prevalent serogroup in Wuping, Changtai, Fuqing, Luoyuan and Jianyang, while Icterohaemorrhagiae was dominant in Pucheng (Table 2).

The seroprevalence was significantly different across species of rodents (*P* < 0.05) (Table S11). The highest MAT seroprevalence was *R. losea* (66/214, 30.84%), followed by *B. indica* (1/4, 25.0%), *R. flavipectus* (11/60, 18.33%), *M. fortis (1/6, 16.67%), N. fulvescens* (10/63,15.87) and *A. agrarius* (1/8, 12.50%).

## Discussion

Leptospirosis is a zoonotic disease caused by pathogenic *Leptospira spp.* and is considered endemic in Fujian Province. The role of rodents as a source of *Leptospira* infection has long been suggested, particularly as rodents are ubiquitous worldwide [1, 12]. However, systematic studies on the primary vector, rodents, and the serological and molecular characteristics of *Leptospira* circulating among rodent populations in Fujian have been limited. In this study, we provided the systematic distribution of rodents, the carrier status of *Leptospira spp.* in rodents by culturing and serological detection, and the molecular characteristics of pathogenic *Leptospira* circulating in potential reservoirs of rodent populations in Fujian Province during 2018–2020, which plays an important role in the epidemiology of leptospirosis.

Four serogroups of Javanica, Icterohaemorrhagiae, Pyrogenes, and Bataviae were identified in these 14 strains isolated from rodents in Fujian, with serogroup Javanica being the most prevalent (7/14, 50.00%). In addition, the serological analysis showed that the seroprevalence was 23.26%, and the four serogroups Javanica, Pomona, Australis, and Icterohaemorrhagiae were found in serum samples in rodent populations by MAT. Among these serogroups, Javanica was the major circulating leptospiral serogroup (79/90, 87.78%). Therefore, Javanica was the most prevalent serogroup in Fujian, which was similar to our previous report in Jiangxi [11]. The lower diversity of leptospirosis serogroups/STs in Fujian may be due to a low number of isolates obtained from the rodent population. In this study, the two most abundant species, *L. interrogan,* and *L. borgpetersenii,* were identified in Fujian, which is consistent with a previous epidemiological investigation of leptospirosis in humans and potential small animal reservoirs in Jiangxi and worldwide [11]. It was reported that four species, *L. borgpetersenii*, *L. interrogan*s, *L. kirschneri* and *L. weilli,* were detected in rodents and human isolates in Thailand, Lao PDR, and Cambodia, with *L. interrogans* and *L. borgpetersenii* widely distributed among rodent populations [13]. It was reported that *L. interrogans, L. kirschneri* and *L. borgpetersenii* were the most widespread and prevalent species detected in Russia [14]. Together, these data revealed that the major *Leptospira* species from these neighbouring countries were similar.

In this study, 6 different STs were obtained from 14 pathogenic *Leptospira* isolates in Fujian Province, with ST143, ST1, and ST17 being the most prevalent STs (Table S2). It was reported that the five most predominant STs ( ST1, ST143, ST105, ST37, and ST17) were identified as long-term and ubiquitous virulent strains throughout Jiangxi Province [11]. ST143, as the most prevalent ST in Fujian and Jiangxi, was also identified in Malaysia [15]. ST17 was also identified in 90 strains of serogroup Icterohaemorrhagiae in Sao Paulo [16]. Five common STs (ST37, ST17, ST199, ST110, and ST146) were reported to have a long-term and ubiquitous distribution in Russia [14]. ST17 was isolated from rodents, pigs, dogs, and humans in Argentina and China [17–19]. ST143 was dominant in rodents in Malaysia and China [18, 20]. Therefore, these prevalent STs (ST17 and ST143) reported in Fujian and Jiangxi in China were also the same prevalent STs in the rest of the world. Generally, there is a complex phylogenetic structure and different distribution of STs of *Leptospira* worldwide. These predominant isolates are likely to have adaptive selective advantages in the environment or potential reservoirs, allowing them to circulate worldwide. These results highlight the importance of understanding the epidemiology and ecology of *Leptospira* worldwide. Further studies are needed to explore the molecular mechanisms that allow them to develop into more prevalent pathogenic strains.

In this study, the density of field rodents was measured to be between 4.81–16.77% at different sampling sites in Fujian Province, which is similar to the rates in Jiangxi Province in China (4.76–12.56%) determined by our previous surveys [11]. However, the most abundantly trapped species differed in these two provinces, with *R. losea, N. fulvescens* and *R. flavipectus* dominating in Fujian and *A. agrarius* and *R. losea* being most common in Jiangxi. The overall carriage rate (14/710, 1.97%) in rodents detected in this investigation based on culture isolation in Fujian was much lower than previous studies based on isolation in other countries such as Malaysia (6.7%) [21], Barbados(16% to 19%) [22], and Brazil (80.3%) [23]. It is important to note that different methods were performed for trapping and determining the presence of leptospires in other labs. The carriage rates of the 6 different species of rodents in Fujian were between 0.71–18.18%. The highest carriage rates based on culture isolation were those of *A. agrarius* (2/11, 18.18%) in Fujian, which is consistent with previous studies showing that *A. agrarius* was the major small animal reservoir in Jiangxi and Guizhou Provinces in China [11, 24].

The infection rates of *Leptospira* in rodents vary in different areas around the world. In this study, the seropositivity of pathogen leptospiral renal carriage in trapped rodents using MAT was 23.26%. It was reported that 8% of 266 rodents were seropositive, primarily for the serovars Australis and Grippotyphosa, in southern Germany [8]. It was reported that 27.1% of *R. norvegicus* were seropositive and that 70% of the positive serum samples reacted with serogroup Bataviae in northern Vietnam, Hanoi, and Bac Giang [25]. It was reported that 68.1% of the 142 captured *R. norvegicus* were seropositive with a MAT titer of ≥ 1:100 in Brazil [23]. The seroprevalence in rats was 7.1% in Andaman Islands [26]. Leptospirosis infection is commonly associated with exposure to contaminated environmental sources, such as those caused during flooding events or high rainfall seasons, by working in rice, pineapple, banana or cane fields, or by exposure to river water during recreational activities or adventure tourism [1, 27], including caving [28], canoeing, rafting, kayaking [29, 30] and triathlons [31]. Our study found that rodents had a relatively high degree of infection with *Leptospira* in Fujian, indicating a potential risk of rodent-borne leptospirosis in this region. Fujian Province is a significant epidemic region for leptospirosis, and rice, banana, cane and tea are the leading local crops. Additionally, Changtai is famous for rafting and kayaking. Therefore, agricultural workers, water sportsmen, and travellers with possible exposures in this epidemic region are at high risk for leptospirosis in Fujian. Leptospirosis ranges in severity from a mild, self-limited febrile illness to a life-threatening illness including renal failure, hypotension, haemorrhage and respiratory failure [27]. The signs and symptoms of leptospirosis are usually mistaken for other causes of an acute febrile syndrome, such as influenza [27]. Unfortunately, people living in Fujian Province have a poor level of knowledge about potential risk factors for leptospirosis. Therefore, increased self-protection awareness and appropriate personal protective measures such as rubber boots, gloves, protective eyewear, or prophylaxis with doxycycline are needed to prevent *Leptospira* infections in Fujian.

In this study, the leptospiral diversity of STs was significantly different across the different species of captured rodents in Fujian. ST143 was distributed in *R. flavipectus* and *R. losea,* and ST1 was distributed in *A. agrarius,* which was consistent with our previous epidemiological investigation of leptospirosis in Jiangxi [18]. In addition, the leptospiral diversity of species/serogroups/STs was significantly different across the six other regions. The leptospiral diversity may be due to the difference in special geographic areas. In this study, Jianyang and Pucheng are located in the northern mountain region. Wuping and Changtai were hills and plain sites, and Luoyuan and Fuqing were eastern coastal areas. The differences in ecological factors, such as altitude, geomorphology, climate among these areas, and various local animal host species may influence leptospiral diversity.

This study revealed the prevalence rates and genetically diverse pathogenic *Leptospira* circulating among rodent populations in Fujian Province, China, suggesting that rodents could be a critical *Leptospira* source. Based on the high infection rate present in this population, these findings highlight the risk of proximity of the infection reservoirs to humans. Furthermore, the various distributions of genotypes of pathogenic *Leptospira* may provide a clue regarding species-specific antigens for vaccine design in different epidemic regions. Therefore, active and long-term monitoring of infections in wild rodents and constructing a suitable database in epidemiological tracing analysis in outbreaks of leptospirosis are necessary. These results may be useful in developing guidelines for the accurate and rapid diagnosis, prevention, and control of leptospirosis in Fujian Province.

## Conclusions

This study detailed a systematic epidemiological investigation, including the molecular characteristics and seroprevalence of pathogenic *Leptospira* in potential reservoirs of rodent populations in Fujian Province during 2018–2020. Two pathogenic species, four serogroups and 6 STs were identified in these 14 isolates by 16S rDNA gene sequencing, MAT, and MLST analysis. Significant geographic variations in the distribution of dominant serogroups, species and STs were found among the *Leptospira* isolates in Fujian. The serological analysis revealed a relatively high seroprevalence rate of leptospirosis among rodent populations in Fujian, indicating a potential risk of rodent-borne leptospirosis in this region. Thus, our present study provides a blueprint for further disease prevention and control.

## Methods

### Rodent collection from Fujian Province

This study and research protocols were approved by the Ethical Committee of Fujian Center for Disease Control and Prevention, China (No: FJCDCNT1811-2015). The trapping, handling, and euthanasia of wild rodents in this study were carried out in accordance with the Guide for the Care and Use of Laboratory Animals (8th edition). Furthermore, this study was carried out in compliance with the ARRIVE guidelines.

Six different trapping sites representing different geographical locations, including Jianyang (27.33 N; 118.12 E) and Pucheng (27.92 N; 118.53 E) (north), Luoyuan (26.48 N; 119.55 E) and Fuqing (25.42 N; 119.23 E) (east), Wuping (25.10 N; 116.10 E) (west), Changtai (24.62 N; 117.75 E) (south), were selected based on the disease incidence in Fujian Province over the past few years.

Rodents were trapped using the trap-night method from April to October in 2018 through 2020 within the framework of the CERoPath project (www.ceropath.org) [32]. Trap placement was based on indicators of rodent activity such as rice field environments, open sewers, tracks, or faeces. Within each sample site, rodents were trapped over an area of approximately 10 square kilometres. Trapping was conducted with peanut bait on the first afternoon and collected in the following early morning during a period of five days and four nights. At each trapping site, 10 trapping lines, including 10 locally hand-made wire traps (approximately 40 × 12 × 12 cm), were placed, with at least five metres of spacing between traps [32]. First, the gender, genus and species of the trapped rodents were identified based on phenotypic characteristics (body, ears, tail, fur colour, and sex) [33]. The rodent density was calculated using the formula: (Number of rodents trapped in each region/Number of total traps successfully placed for each region * 100).

### Leptospiral isolation from Fujian Province and DNA extraction from *Leptospira* isolates

After transportation to the laboratory, the rodents were euthanized by isoflurane inhalation, and kidney tissue was collected following the Guide for the Care and Use of Laboratory Animals (8th edition). For the isolation of leptospires, approximately 1 g of fresh kidney tissue samples from rodents were aseptically inoculated into 10 ml of liquid Ellinghausen-McCullough-Johnson-Harris (EMJH) medium (Difco Laboratories, USA) with 5-fluorouracil (Merck, Germany) at 28 °C in the laboratory. Within 48 h, 1 mL of the initial tube was transferred into a second EMJH culture bottle at a dilution of approximately 1:100 to reduce the inhibitory effects of anaerobic conditions created by autolysis of the tissues. The cultures were observed weekly using dark-field microscopy to determine the presence of *Leptospira* for up to 3 months. Positive cultures were immediately subcultured in fresh liquid EMJH medium. Samples with no growth of *Leptospira* after 3 months were considered negative [34]. The isolates were stored long-term at − 70 °C and were passaged every six months. When needed, the isolated colonies were subcultured at 28 °C in EMJH liquid medium for 7 days and DNA was extracted from the cultures of *Leptospira* isolates using Wizard Genomic DNA Purification Kit (Promega, Madison, USA) according to the manufacturer’s directions.

### Species identification of pathogenic *Leptospira* strains isolated in Fujian Province

Species identification of the isolates was conducted based on 16S rDNA gene sequencing with lengths ranging from 1,422 to 1,432 bp for leptospires as previously described by Morey [35]. A total of 68 reference sequences representing the major reported pathogenic, intermediate and saprophytic *Leptospira* species worldwide were obtained from the GenBank database (Table S12) [35–38]. The 16S rDNA gene sequences of *Turneriella parva* NCTC 11395 T and *Leptonema illini* NCTC 11301 T were set as the outgroup (Table S12) [35]. The 16S rDNA gene sequences from leptospiral isolates from Fujian were compared with the 68 reference sequences, and phylogenetic analysis was performed using Clustal W. A neighbor-joining tree was constructed using Mega software version 6.00 with a bootstrap value of 1,000 replicates.

### Serogroup identification of leptospiral strains isolated from Fujian Province

MAT was performed for serogroup identification of the leptospiral isolates by a panel of 15 Chinese prevalent serogroup-specific rabbit antisera representing 15 serogroups provided by the National Institutes of Food and Drug Control, China (Table S13). All reference rabbit antisera were serially diluted with a starting dilution of 1: 50 and up to a dilution of 1: 6, 400 in sterile saline using twofold dilutions and tested with leptospiral isolate suspensions. MAT endpoint titers were determined until the last well showing 50% agglutination in free-moving leptospires was observed. The serogroup scoring the highest MAT titer was defined as the corresponding serogroup.

### MLST analysis of isolated leptospiral strains

MLST analysis of the isolates was conducted using seven housekeeping genes (*glmU*, *pntA, sucA, tpiA*, *pfkB*, *mreA* and *caiB*) as previously described [39]. The available PubMLST *Leptospira* database (http://pubmlst.org/leptospira/) was used for assigning alleles and STs. Phylogenetic analysis was conducted using the unweighted pair group method with average linkages provided in BioNumerics software version 5.10.

### Serological antibody detection in rodents using MAT.

Serum samples from the trapped rodents were collected, and MAT analyses were performed for *Leptospira*-specific antibodies with a panel of 10 live pathogenic *Leptospira* antigens representing 10 different local serogroups (Icterohaemorrhagiae, Javanica, Autumnalis, Australis, Pomona, Hebdomadis, Grippotyphosa, Pyrogenes, Bataviae and Sejroe) as previously described [40]. All sera were serially diluted with a starting dilution of 1: 10 and reaching a screening dilution of 1: 80 in sterile saline using twofold dilutions and tested against antigen suspensions to establish the antibody titer. Following incubation at 30 °C for 2 h, each suspension was observed by a dark field microscope (Nikon, Eclipse 50i) at 400X magnification. The sera with positive screening results (1:160) were further diluted to determine the endpoint titer for each sample. The MAT endpoint titer was defined as the highest dilution showing ≥ 50% agglutination in free-moving leptospires [41] compared to a negative control obtained by using sterile saline instead of serum. Sera were considered positive for seroreactivity at a MAT titer ≥ 1:160 against at least one serovar [42].

### Statistical analysis

Fisher’s exact test was performed to compare the differences in the distribution of dominant *Leptospira* across species, serogroups and STs between rodent species and six different collected sites. In addition, the statistical chi-square test was used to determine if there were differences in *Leptospira* positivity rates between different collected sites and rodent species. The p value was computed by Monte Carlo simulation. All statistical analyses were conducted using R software (R version 3.5.1, https://www.r-project.org/) [43], considering a significance level of 0.05.

## Supplementary Information

**Additional file 1: Table S1.** Species distribution of the captured rodents among the six different regions in Fujian. (XLS). **Table S2.** Fourteen pathogenic *Leptospira* strains isolated from Fujian Province were used in this study. (XLS). **Table S3.** Species distribution of the 14 pathogenic *Leptospira* strains among the six different regions in Fujian. (XLS).** Table S4.** Species distribution of the 14 pathogenic *Leptospira* strains among the different species of captured rodents in Fujian. (XLS).** Table S5.** Serogroup distribution of the 14 pathogenic *Leptospira* strains among the six different regions in Fujian. (XLS). **Table S6.** Serogroup distribution of the 14 pathogenic *Leptospira* strains across the different species of rodents in Fujian. (XLS). **Table S7.** Sequence type distribution of the 14 pathogenic *Leptospira* strains among the six different regions in Fujian. (XLS). **Table S8.** Sequence type distribution of the 14 pathogenic *Leptospira* strains among the different species of rodents in Fujian. (XLS). **Table S9.** Results of serological antibody detection using MAT. (XLS). **Table S10.** Seroprevalence between the six different regions in Fujian Province. (XLS). **Table S11.** Seroprevalence between the ten different species of rodents. (XLS). **Table S12.** 16S rDNA gene sequences of 68 *Leptospira* reference species, *Turneriella parva* NCTC 11395 T and *Leptonema illini* NCTC 11301 T obtained from the GenBank database. (XLS). **Table S13.** Detailed information on 15 Chinese prevalent serogroup-specific rabbit antisera.

## Data Availability

The data generated and/or analyzed during the current study are available from the corresponding author on request.

## Abbreviations

MLST: Multilocus sequence typing
MAT: Microscopic agglutination test
